# Mechanofluorochromism (MFC) of Donor*–π*–Acceptor (D*–π*–A)‐Type Fluorescent Dyes

**DOI:** 10.1002/tcr.202500211

**Published:** 2025-12-02

**Authors:** Yousuke Ooyama

**Affiliations:** ^1^ Applied Chemistry Program, Graduate School of Advanced Science and Engineering Hiroshima University Higashi‐Hiroshima Japan

**Keywords:** *π*–*π* interaction, D–*π*–A fluorescent dye, dipole–dipole interaction, intramolecular charge transfer, mechanofluorochromism

## Abstract

Mechanofluorochromism (MFC) is a photophysical phenomenon in which the color and fluorescent color of solid‐state organic or metal complex fluorescent dyes change upon external mechanical stimulation (grinding) and recover to their original ones upon heating or exposure to solvent vapor. We discovered that newly developed donor*–π*–acceptor (D*–π*–A) fluorescent dyes exhibit bathochromic or hypsochromic‐shifted MFC (b‐MFC or h‐MFC). This MFC arises from reversible switching between the crystalline and amorphous states, accompanied by changes in dipole–dipole and intermolecular *π* *–π* interactions upon grinding and heating. Indeed, such MFC not only is of a great scientific interest in photochemistry and photophysics but also has great potential for development of smart materials for next‐generation optoelectronic devices, including rewritable photoimaging and electroluminescence devices. In this Personal Account, we offer an insight into the mechanism for the expression of MFC and present molecular design directions for creating D*–π*–A‐type mechanofluorochromic dyes which can exhibit b‐MFC or h‐MFC.

## Graphical abstract

Mechanofluorochromism (MFC) is a photophysical phenomenon that induces changes in fluorescent colors as well as colors of organic fluorescent dyes in the solid‐state by external mechanical stimuli. In this Personal Account, we offer an insight into the mechanism for the expression of MFC and present a direction in the molecular design toward creating D*–π*–A‐type mechanofluorochromic dyes.

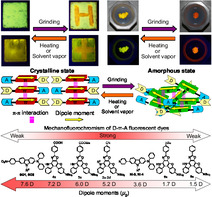

## 1 Introduction

**FIGURE 1:**
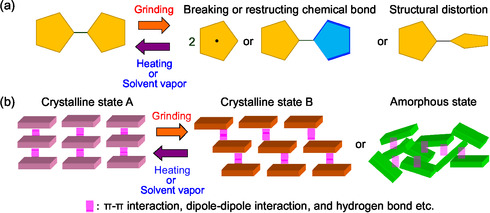
(a) Intra‐MFC due to changes in the chemical structures of dye molecules (breaking and restructuring chemical bonds and structural distortion). (b) Inter‐MFC due to changes of molecular orientation and arrangement with solid‐state transformation (crystalline‐to‐crystalline and crystalline‐to‐amorphous phase transitions).

Mechanofluorochromism (MFC) is the phenomenon in which the color and fluorescence of solid‐state organic or metal complex fluorescent dyes change upon external mechanical stimuli (e.g., grinding) and then revert to their original state upon heating of the ground solids or exposure of it to solvent vapor [1, 2, 3, 4, 5, 6, 7, 8–9]. Generally, MFC occurs due to reversible changes in the electronic structure of dye molecules and/or intermolecular interactions between them. Thus, these changes by grinding, heating, or exposure to solvent vapors are triggered by changes in the chemical structures of dye molecules (breaking and restructuring chemical bonds and structural distortion) or changes in molecular orientation and arrangement with solid‐state transformation (crystalline‐to‐crystalline and crystalline‐to‐amorphous phase transitions), resulting in changes in the photoabsorption and fluorescence properties. Intramolecular MFC (intra‐MFC) which occur due to changes in chemical structure of dye molecules (e.g., lophine dimer, bianthrone, and spiropyran derivatives) by breaking and reconstructing chemical bonds during the grinding–heating process is liable to exhibit poor reversibility, because the chemical structure after grinding is unstable, potentially causing side reactions (Figure 1) [10, 11, 12–13]. Unlike the intra‐MFC, intermolecular MFC (inter‐MFC), due to reversible changes of molecular orientation and arrangement of dye molecules, makes it more durable and suitable and is expected to lead to the creation of innovative rewritable optical imaging and recording devices [14, 15, 16, 17, 18, 19, 20, 21, 22, 23, 24, 25, 26, 27, 28, 29, 30, 31, 32, 33, 34, 35, 36, 37, 38, 39, 40, 41, 42, 43, 44, 45, 46, 47, 48, 49, 50, 51, 52, 53, 54, 55, 56, 57, 58, 59, 60, 61, 62, 63, 64, 65, 66, 67, 68, 69, 70, 71, 72, 73, 74, 75, 76, 77, 78, 79, 80, 81, 82, 83, 84, 85, 86, 87, 88–89]. However, there have been few efforts to achieve reversible changes in the solid‐state fluorescence properties of dyes by controlling their molecular orientation and arrangement through grinding and heating process, and thus, the detailed mechanism of such dynamic solid‐state luminescence characteristics remains unexplained. The reason is that it has not been possible to express MFC in a series of fluorescent dyes, making the systematic experimental and theoretical investigation difficult. Therefore, it is necessary to provide molecular design directions for creating mechanofluorochromic dyes, leading to comprehensive elucidation of inter‐MFC.

## 2 Research Highlights

**FIGURE 2:**
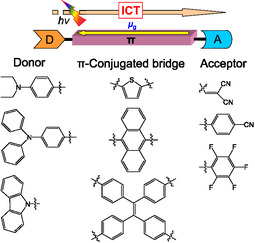
Schematic representation of D*–π*–A‐type fluorescent dyes.

Among various types of fluorescent dyes, donor*–π*–acceptor (D*–π*–A) fluorescent dyes are constructed from an electron‐donating moiety (D) and an electron‐withdrawing moiety (A) connected by a *π*‐conjugated bridge and exhibit strong photoabsorption and fluorescence emission characteristics originating from intramolecular charge transfer (ICT) excitation from the D moiety to the A moiety (Figure 2) [42, 44, 57, 63, 88, 89–90]. Thus, the dipole moments as well as the optical and electrochemical properties of D*–π*–A‐type fluorescent dyes can be tuned by altering the magnitude of the electron‐donating ability of D moiety and the electron‐withdrawing ability of A moiety and the electronic characteristics of the *π*‐conjugated bridge. Meanwhile, D*–π*–A‐type fluorescent dyes exhibit significant fluorescence quenching (aggregation‐induced quenching: ACQ) and bathochromic shifts in the photoabsorption and fluorescence maxima in the solid state compared to their solution state, which are attributed to the delocalization of excitons or excimers through the formation of intermolecular *π* *–π* interactions between dye molecules in the solid state. However, by varying the steric hindrance of the D and A moieties and/or the substituents on the *π*‐conjugated bridge, it is possible to control not only the intermolecular dipole–dipole and *π* *–π* interactions but also the molecular orientation and arrangement in the solid state.

Our team have discovered for the first time that crystals of a new series of D*–π*–A‐type fluorescent dyes reversibly change both their color and fluorescent color upon grinding and heating (or exposure to solvent vapor) and have termed this phenomenon mechanofluorochromism (MFC) [69, 70, 71, 72, 73, 74–75]. However, few methods and techniques have been established to systematically investigate and evaluate the MFC properties of organic dyes that are caused by changes in molecular orientation and arrangement. Hence, we demonstrated that the presence or absence of MFC can be evaluated by the photoabsorption, fluorescence excitation, fluorescence spectral measurements, fluorescence quantum yields, time‐resolved fluorescence measurements, differential scanning calorimetry (DSC), X‐ray powder diffraction (XRD), single‐crystal X‐ray diffraction, and density measurements for the solids before and after grinding and after heating of the ground solids and molecular orbital (MO) calculations.

In this Personal Account, we present molecular design of a new series of D*–π*–A‐type fluorescent dyes with controlled molecular orientation and arrangement and intermolecular interactions suitable for elucidating the inter‐MFC and offer a direction in the molecular design toward creating D*–π*–A‐type mechanofluorochromic dyes which can exhibit a bathochromic or hypsochromic shift‐type MFC (b‐MFC or h‐MFC), based on the experimental and theoretical insight into the mechanism for the expression of MFC.

## 3 MFC of Heteropolycyclic D–*π*–A‐Type Fluorescent Dyes

**FIGURE 3:**
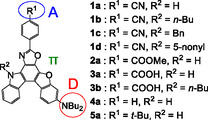
Chemical structures of heteropolycyclic D*–π*–A‐type fluorescent dyes **1a**–**1d** and **2a**–**5a**.

We serendipitously discovered that a newly developed heteropolycyclic D*–π*–A‐type fluorescent dye **1a**, which has diphenylamino group as a D moiety and *p*‐cyanophenyl group as an A moiety connected by benzofuro[2,3‐*c*]oxazolo[4,5‐*a*]carbazole skeleton as a *π*‐conjugated bridge, shows inter‐b‐MFC; grinding of as‐recrystallized dyes by using a spatula induces a bathochromic shift of the photoabsorption maximum wavelength (*λ*_max_^abs‐solid^) and the fluorescence maximum wavelength (*λ*_max_^fl‐solid^) and an increase in the fluorescence quantum yield (Φ_fl_‐_solid_), and subsequent heating of the ground solids at ca. 120°C or exposure of it to solvent vapors such as acetone restores the original color and fluorescent color (Figure 3) [69–71]. In order to clarify the effect of the dye molecular structure on the expression of inter‐MFC by changing the molecular orientation and arrangement and intermolecular interactions, we designed and developed **1b**–**5a** and **3b** with *p*‐substituted phenyl groups with different electron‐withdrawing ability (*R* ^1^) and substituents (*R* ^2^) with different bulkiness introduced on the nitrogen atom of the carbazole ring. The electron‐withdrawing ability of *p*‐substituted phenyl groups increases in the order of **5a** (*R* ^1^ = *t*‐Bu) < **4a** (*R* ^1^ = H) ≪ **3a**, **3b** (*R* ^1^ = COOH) < **2a** (*R* ^1^ = COOMe) < **1a** (*R* ^1^ = CN), and steric bulkiness of *R* ^2^ increases in the order of **1a** (*R* ^2^ = H) < **1b** (*R* ^2^ = *n*‐Bu) < **1c** (*R* ^2^ = Bn) < **1d** (*R* ^2^ = 5‐nonyl). Therefore, the dyes **1a**–**1d**, **2a**, **3a**, and **3b** have the strong electron‐withdrawing substituents, whereas the dyes **4a** and **5a** do not. Specifically, the structural features of this D*–π*–A‐type fluorescent dye are as follows: (1) Its electron‐donating dialkylamino group (D) and electron‐withdrawing (A) substituent (*R* ^1^) are linked by a heteropolycyclic *π*‐conjugation, enabling strong photoabsorption and fluorescence characteristics due to ICT from the D moiety to the A moiety; (2) the magnitude of the dipole moment can be adjusted by changing the substituent *R* ^1^, allowing the control of the dipole–dipole interaction in the solid state; and (3) *π* *–π* stacking between dye molecules in the solid state is adjustable by varying the steric hindrance of substituent *R* ^2^.

**FIGURE 4:**
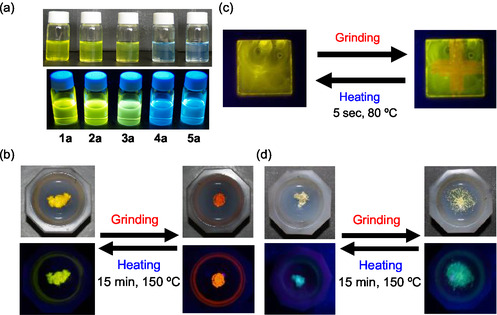
(a) Photographs of **1a**–**5a** in 1,4‐dioxane under room light (top) and under UV‐light irradiation (down). Photographs of (b) solid **1b** under room light (top) and under UV irradiation (down), of (c) **1b**‐cast film under UV irradiation, and of (d) solid **5a** under room light (top) and under UV irradiation (down) before and after grinding of as‐recrystallized dyes after heating of the ground solid. Adapted with permission from refs. [69–71]. Copyright 2009 John Wiley & Sons, Inc. Copyright 2011 Royal Society of Chemistry. Copyright 2010 Elsevier.

All the dyes showed a *λ*_max_^abs‐solution^ at 387–430 nm (*ε*_max_ = 23 200–32 900 M^–1^ cm^–1^), which is originated from ICT excitation characteristics from the dibutylamino group to the *p*‐substituted phenyl group. In the corresponding fluorescence spectra, the *λ*_max_^fl‐solution^ of all the dyes appeared at 455–539 nm. Indeed, the *λ*_max_^abs‐solution^ and *λ*_max_^fl‐solution^ of the dyes **1a**–**5a** appeared in a longer wavelength region in the order of **5a** (387 and 455 nm) < **4a** (388 and 463 nm) < **3a** (416 and 526 nm) < **2a** (418 and 532 nm) < **1a** (428 and 539 nm), which is consistent with the order of the strength of electron‐withdrawing ability of the substituent *R* ^1^. The photoabsorption and fluorescence spectra of the dyes **1a**–**1d** were also similar to each other, indicating that the effect of the *N*‐alkylation (*R* ^2^) of the carbazole ring has only a small effect on the optical properties of the dyes. Furthermore, all the dyes exhibit very strong fluorescence property with a fluorescence quantum yields (Φ_fl_‐_solution_) of nearly unity. The time‐resolved fluorescence measurements revealed that the decay profiles are well fitted to a single exponential function with the fluorescence lifetime (*τ*_f_‐_solution_) of 2.7–4.3 ns for **1a**–**1d**, **2a**–**5a**, and **3b**. The solution colors of the dyes in 1,4‐dioxane are greenish yellow for **1a**–**1d**, **2a**, **3a**, and **3b** and light blue for **4a** and **5a** (Figure 4a). The fluorescence colors under UV lamp (365 nm) were yellow for **1a**–**1d**, **2a**, **3a**, and **3b** and blue for **4a** and **5a**.

**FIGURE 5:**
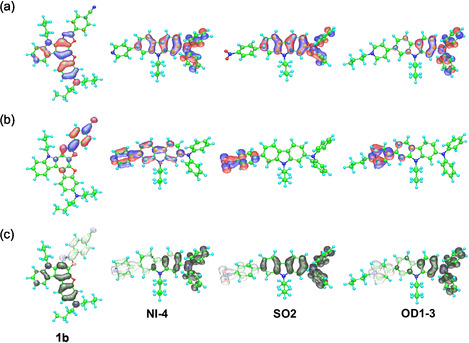
(a) HOMO and (b) LUMO of **1b**, **NI‐4**, **SO2**, and the dye cation **OD1‐3** (without counter anion) derived from MO calculations. The blue and red lobes represent the negative and positive signs of the MO coefficients. The magnitude of each lobe corresponds to the MO coefficient. (c) Calculated electron density changes accompanying the first electronic excitation of **1b**, **NI‐4**, **SO2**, and the dye cation **OD1‐3** (without counter anion). The red and blue lobes represent the increase and decrease in electron density accompanying the electronic transition, respectively. The area of the lobe indicates the magnitude of the electron density change. Adapted with permission from refs. [70, 72, 73, 75]. Copyright 2012 John Wiley & Sons, Inc. Copyright 2011 Royal Society of Chemistry. Copyright 2012 and 2013 Elsevier.

The MO calculations indicated that for all the dyes, the highest occupied molecular orbital (HOMO) is distributed over the benzofuro[2,3‐*c*]oxazolocarbazole skeleton containing dibutylamino group, and the lowest unoccupied molecular orbital (LUMO) is mainly localized at the *p*‐substituted phenyl groups (Figure 5). Furthermore, the change in charge density accompanying the first electron transition suggested that all the dyes exhibit strong ICT characteristics from the benzofuro[2,3‐*c*]oxazolocarbazole skeleton containing dibutylamino group to the *p*‐substituted phenyl group upon photoexcitation. The dipole moments (*μ*_g_) of the dyes in the ground state increased in the following order: **4a** (1.47 Debye) < **5a** (1.65 Debye) < **1a**–**1d** (5.13–5.21 Debye) < **2a** (6.03 Debye) < **3a** (7.18 Debye) < **3b** (7.59 Debye). This result indicates that the *μ*_g_ values of **1a**–**3a** and **3b**, which have a strong electron‐withdrawing group *R* ^1^, are approximately 3–5 times larger than that of **4a** and **5a**.

**FIGURE 6:**
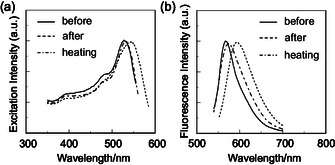
(a) Solid‐state excitation at *λ*_max_^fl‐solid^ and (b) fluorescence spectra (*λ* ^ex^ = *λ*_max_^ex‐solid^) of solid **1d** before and after grinding of as‐recrystallized dye and after heating of the ground solid at 150°C. Adapted with permission from ref. [69]. Copyright 2009 John Wiley & Sons, Inc.

The colors of the as‐recrystallized dyes are yellow for **1a**–**1c** and **2a**, yellowish orange for **1d**, **3a**, and **3b**, and light yellow for **4a** and **5a**. The fluorescence colors under UV lamp (365 nm) are yellowish green for **1a**–**1d**, **2a**, **3a**, and **3b**, green for **4a**, and bluish green for **5a** (Figure 4b,c). The solid‐state fluorescence excitation and fluorescence spectral measurements showed that the excitation maximum wavelengths (*λ*_max_^ex‐solid^) and fluorescence maximum wavelengths (*λ*_max_^fl‐solid^) of the crystals **1a**–**5a** and **3b** appeared at 443–531 and 471–571 nm, respectively, which were bathochromically shifted by ca. 55–115 and 15–40 nm, respectively, compared to the *λ*_max_^abs‐solution^ and *λ*_max_^fl‐solution^ in 1,4‐dioxane (Figure 6). Furthermore, the Φ_fl_‐_solid_ values of **1a**–**1d**, **2a**–**5a**, and **3b** in the crystalline state are 0.02–0.15, which are significantly lower than the Φ_fl_‐_solution_ values in 1,4‐dioxane. It is known that the bathochromic shifts of *λ*_max_^abs^ and *λ*_max_^fl^ and the decrease in Φ_fl_ values of D*–π*–A‐type fluorescent dyes upon transition from solution to the crystalline state would be due to the delocalization or deactivation of excitons by the formation of *π* *–π* interactions and intermolecular hydrogen bonds between dye molecules in the crystals [91, 92–93]. In particular, the bathochromic shift of *λ*_max_^abs^ and *λ*_max_^fl^ and the decrease in Φ_fl_ value of **3a** upon transition from solution to the crystalline state are significantly larger than those of **1a**–**1d** and **2a**, **4a**, and **5a**. Presumably, this is caused by the formation of intermolecular hydrogen bonds between the carboxyl groups of **3a** in the crystalline state. However, the Φ_fl_‐_solid_ value (0.04) of **3b** is higher than that of **3a** (Φ_fl_‐_solid_ ∼ 0). The fact demonstrates that the introduction of a butyl group on the nitrogen atom of carbazole ring effectively suppresses the *π* *–π* interactions between dye molecules. These results indicate that the *N*‐alkylation of carbazole ring effectively suppresses the intermolecular *π* *–π* interaction between dye molecules that cause fluorescence quenching in the solid state.

To investigate the MFC of the heteropolycyclic D*–π*–A‐type fluorescent dyes, as‐recrystallized dyes **1a**–**1d**, **2a**–**5a**, and **3b** were ground in an agate mortar and pestle with a stress of 50–100 N/cm^2^. By grinding the crystals of **1a**–**1d**, **2a**, and **3b**, their color changed from yellow to orange, and their fluorescence color changed from yellowish green to reddish orange (Figure 4b,c). However, the color and fluorescence color of crystal of **4a** and **5a** changed little by grinding. The solid‐state excitation and fluorescence spectral measurements showed that when the as‐recrystallized dyes were ground, the *λ*_max_^ex‐solid^ and *λ*_max_^fl‐solid^ for **1a**–**1d** are bathochromically shifted by 21–54 and 25–53 nm, respectively, indicating expression of the b‐MFC (Figure 6). In addition, the magnitude of the bathochromic shift (Δ*λ*_max_^ex‐solid^ and Δ*λ*_max_^fl‐solid^) decreases in the order of **1a** (54 and 53 nm) > **1b** (47 and 53 nm) > **1c** (36 and 47 nm) > **1d** (21 and 25 nm), which is consistent with the increasing order of steric hindrance of substituent *R* ^2^. Meanwhile, the *λ*_max_^ex‐solid^ and *λ*_max_^fl‐solid^ for **2a**, **3a**, **4a**, and **4b** are bathochromically shifted by 15–40 and 18–41 nm, respectively, by grinding. Furthermore, the degrees (Δ*λ*_max_^ex‐solid^ and Δ*λ*_max_^fl‐solid^) of the bathochromic shift of *λ*_max_^ex‐solid^ and *λ*_max_^fl‐solid^ for **1a**–**4a** after grinding increase in the order of **4a** (15, 18 nm) < **3a** (37, 32 nm) < **2a** (40, 41 nm) < **1a** (54, 53 nm), agreeing with the order of increasing the electron‐withdrawing ability of the substituent *R* ^1^. Interestingly, the Φ_fl_‐_solid_ values of **1a**–**1d**, **2a**, **4a**, and **3b** increased by about 1.5–3 times by grinding. On the other hand, the crystals of **5a**, which has a *t*‐butyl group as substituent *R* ^1^, showed almost no change in the *λ*_max_^ex‐solid^ and *λ*_max_^fl‐solid^ by grinding and thus did not show MFC (Figure 4b). Furthermore, when the ground solids were heated at 80ºC–230ºC (beyond the recrystallization temperature *T*_c_, as described later) or exposed to solvent vapor such as acetone, the color and fluorescent color of the dyes except **3a** restored to the original ones. In addition, the dye **1d**, which has 5‐nonyl group as a bulky substituent *R* ^2^, showed good reversibility of the solid‐state excitation and fluorescence spectra by grinding and heating and thus exhibited excellent MFC (Figure 6). The time‐resolved fluorescence measurements of all the dyes except **3a** indicated that the fluorescence decay profiles are fitted to biexponential curve with fluorescence lifetimes of *τ*_1‐solid_ = 0.3–1.3 ns and *τ*_2‐solid_ = 1.6–5.9 ns both before and after grinding. The ratios of fractional contributions (*A*_1_/*A*_2_) of **1a**–**1d**, **2a**, and **5a** decreased in common by grinding. Consequently, the fluorescence decay profiles revealed the existence of two distinct emission states in the dye solids before and after grinding.

**FIGURE 7:**
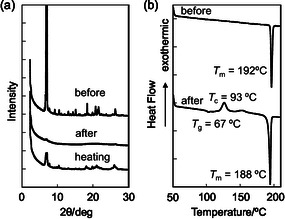
(a) XRD patterns of **1d** before (as‐recrystallized) and after grinding of as‐recrystallized dye and after heating of the ground solid. (b) DSC curves (heating process with scan rate of 10°C min^**−**1^) of **1d** before and after grinding (*T*_g_: glass transition, *T*_c_: crystallization, and *T*_m_: melting). Adapted with permission from ref. [69]. Copyright 2009 John Wiley & Sons, Inc.

**FIGURE 8:**
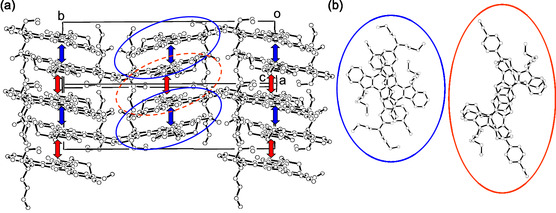
Crystal packing of **1d** (CCDC Deposition Number 681490): (a) stereoview of molecular packing structure and (b) top view of pairs of dye molecules. The up–down arrows denote the intermolecular *π* *–π* interactions. The blue solid and red dotted circles show the *π*‐staking arrangements. Adapted with permission from ref. [69]. Copyright 2009 John Wiley & Sons, Inc.

The XRD measurements of as‐recrystallized dyes **1a**–**1d**, **2a**–**5a**, and **3b** exhibited diffraction peaks assignable to well‐defined microcrystalline structures (Figure 7a). They almost disappeared after grinding, indicating that the ground solids were in an amorphous state with reduced molecular orientation and arrangement. The DSC analysis indicated that the as‐recrystallized dyes **1a**, **1b**, **1d**, **2a**, **4a**, **5a**, and **3b** showed only one sharp endothermic peak due to melting (*T*_m_) (Figure 7b). In contrast, the DSC trace of as‐recrystallized dye **1c** showed an endothermic peak at 253.5°C due to the *T*_m_, an exothermic peak at 258.1°C due to the *T*_c_ associated with the transition to the stable form, and an endothermic peak at 270.0°C due to the melting of the stable form, indicating a typical thermal behavior of polymorphic crystal. On the other hand, the DSC trace for the ground solids of the dyes except **3a** (decomposition without melting) showed an endothermic glass transition (*T*_g_) at around 65°C–170°C and then an exothermic *T*_c_ at around 80°C–220°C before an endothermic *T*_m_ at around 200°C–330°C, indicating typical thermal behavior of amorphous solids. Indeed, the XRD diffraction patterns of the solids after heating of the ground solids beyond *T*_c_ were very similar to those before grinding, suggesting that the microcrystalline structure was restored by heating. Interestingly, the dye **5a** without exhibiting MFC was found to change from fine crystalline state before grinding to the amorphous state after grinding, similar to the dyes **1a**–**1d**, **2a**, **4a**, and **3b** exhibiting MFC. Furthermore, in order to investigate the change in the molecular packing of the solids, the densities of the solids of the dyes **1a**–**1c**, **2a**, and **4a** before and after grinding were estimated using the Archimedean method. It was found that the densities increase from 1.32, 1.22, 1.23, 1.37, and 0.96 g cm^−3^ to 1.36, 1.39, 1.43, 1.41, and 1.01 g cm^−3^ for **1a**–**1c**, **2a**, and **4a**, respectively, by grinding, indicating that after grinding, the dye molecules were more densely packed. Therefore, the XRD and DSC studies and density measurements strongly suggest that the b‐MFC of heteropolycyclic D*–π*–A fluorescent dyes is not solely due to events arising from the reversible change between the crystalline and amorphous states upon grinding and heating, but rather results from the reversible change of the intermolecular interactions between the dye molecules. Indeed, single‐crystal X‐ray structure analysis of **1d** showed that the dye molecules are arranged in two stacking patterns by overlapping *π* planes in an inversion symmetric manner, forming continuous intermolecular *π* *–π* interactions between the dye molecules (Figure 8).

**FIGURE 9:**
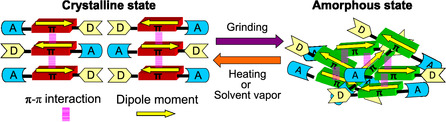
Mechanisms of b‐MFC observed in D*–π*–A‐type fluorescent dyes. Adapted with permission from ref. [69]. Copyright 2009 John Wiley & Sons, Inc.

Therefore, based on the above experimental and theoretical MO calculation results, we have considered the mechanism for the expression of b‐MFC of the heteropolycyclic D*–π*–A‐type fluorescent dye. In the crystalline state, the long‐range intermolecular *π* *–π* interactions between neighboring molecular dyes result in stacking of the dye molecules, where their dipole moments are arranged in an antiparallel direction, maximizing the intermolecular dipole–dipole interaction, leading to a bathochromic shift of *λ*_max_^abs^ and *λ*_max_^fl^ upon transition from solution to the crystalline state. Furthermore, the decrease in Φ_fl_ value upon transition from solution to the crystalline state is ascribable to the deactivation or delocalization of excitons associated with the formation of long‐range intermolecular *π* *–π* interactions between dye molecules in the crystalline state, resulting in a nonradiative decay pathway of the excited state [91–93]. It is also suggested that the two types of dye stacking patterns observed in the crystal structure of **1d** are related to the existence of two fluorescent emission components with different lifetimes (*τ*_1‐solid_ and *τ*_2‐solid_) observed in the solid state (Figure 8). On the other hand, when the as‐recrystallized dyes were ground, the dye molecules approach each other, maximizing the dipole–dipole interaction strength and intermolecular *π* *–π* interaction strength. This is evident from the increased density of the dye molecules in the amorphous state. The strength of dipole–dipole interaction increases with dyes having larger dipole moments. Indeed, the degree (Δ*λ*_max_^ex‐solid^ and Δ*λ*_max_^fl‐solid^) of bathochromic shift of *λ*_max_^ex‐solid^ and *λ*_max_^fl‐solid^ before and after grinding of the dye depends on the electron‐withdrawing ability of the substituent *R* ^1^, and thus, *μ*_g_ values of **1a**–**1d** (5.13–5.21 Debye), **2a** (6.03 Debye), **3a** (7.18 Debye), and **3b** (7.59 Debye) are larger than those of **4a** (1.47 Debye) and **5a** (1.65 Debye). Thus, the bathochromic shift of *λ*_max_^ex‐solid^ and *λ*_max_^fl‐solid^ of **1a**–**1d**, **2a**, **3a**, and **3b** upon transition from the crystalline state to the amorphous state is attributed to the fact that in the amorphous state, the distance between the dye molecules is shortened due to the strong dipole–dipole interactions, resulting in that the dye packing becomes denser than the case of the crystalline state (Figure 9). On the other hand, the nonobvious b‐MFC for **4a** and **5a** is attributed to the small change in dipole–dipole interaction between the crystalline state and the amorphous state due to their small dipole moments. Meanwhile, the Φ_fl_‐_solid_ values in the amorphous state are larger than those in the crystalline state, indicating that, unlike the case of long‐range intermolecular *π* *–π* interactions between dye molecules in the crystalline state, the short‐range intermolecular *π* *–π* interactions between the dye molecules in the amorphous state suppress the deactivation or delocalization of excitons. Furthermore, the b‐MFC of **1a**–**1d** depend on the bulkiness of the substituent *R* ^2^, indicating that the steric factors of the substituent affect the degree of change in intermolecular *π* *–π* interactions between the crystalline state and the amorphous state. Consequently, this study clarified that the b‐MFC of heteropolycyclic D*–π*–A‐type fluorescent dyes are due to the reversible changes in dipole–dipole interactions and intermolecular *π* *–π* interactions between the dye molecules in the crystalline state before grinding and the amorphous state after grinding and offered that the color and fluorescence color change of the D*–π*–A fluorescent dye in b‐MFC can be precisely controlled by adjusting the electron‐donating ability of D moiety, the electron‐accepting ability of A moiety, the steric size of the substituents, and the magnitude of donor–acceptor *π*‐conjugated system. Furthermore, it was suggested that the expression of b‐MFC requires a D*–π*–A‐type fluorescent dye structure with a dipole moment of ca. 5 Debye.

## 4 MFC of Carbazole‐Based D–*π*–A‐Type Fluorescent Dyes

**FIGURE 10:**
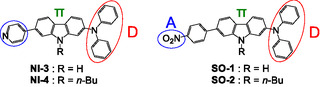
D*–π*–A‐type fluorescent dyes **NI‐3**, **NI‐4**, **SO1**, and **SO2**.

To further explore a direction in the molecular design toward creating D*–π*–A‐type mechanofluorochromic dyes, we designed and developed the carbazole‐based D*–π*–A‐type fluorescent dyes **NI‐3** and **NI‐4** composed of a diphenylamino group as D moiety, a *p*‐pyridine ring as A moiety, and a carbazole skeleton as *π*‐conjugated bridge (Figure 10) [72]. In addition, **NI‐4** has a *n*‐butyl group on the nitrogen atom of the carbazole skeleton.

In 1,4‐dioxane, the dyes **NI‐3** and **NI‐4** showed an *λ*_max_^abs‐solution^ at around 305 nm (*ε*_max_ = 15 700 and 18 000 M^–1^ cm^–1^, respectively) due to the *π* → π* transition, and a photoabsorption band at around 375 nm (*ε*_max_ = 30 200 and 33 000 M^–1 ^cm^–1^, respectively) due to the ICT excitation characteristics from D moiety (diphenylamino group) to A moiety (*p*‐pyridine ring). In the corresponding fluorescence spectra, the *λ*_max_^fl‐solution^ of both dyes appeared at around 420 nm, and the Φ_fl_‐_solution_ value was ca. 0.8. The time‐resolved fluorescence measurements revealed that the fluorescence decay profile of both **NI‐3** and **NI‐4** were well fitted to a single exponential function with *τ*_f_ = 1.9 ns. The solution colors of both dyes in 1,4‐dioxane are nearly colorless, and their fluorescent colors are blue under UV lamp (365 nm). This result indicates that the *N*‐alkylation of the carbazole ring has only a negligible effect on the optical properties of the dye itself.

The MO calculations indicated that for **NI‐3** and **NI‐4**, the HOMO is distributed over the diphenylamino–carbazole moiety and the LUMO is mainly localized on the pyridinyl–carbazole moiety (Figure 5). Furthermore, the change in charge density accompanying the first electron transition suggested that both the dyes exhibit strong ICT characteristics from the diphenylamino–carbazole moiety to the pyridinyl group upon photoexcitation. The *μ*_g_ values of **NI‐3** and **NI‐4** in the ground state were ca. 3.6 Debye.

**FIGURE 11:**
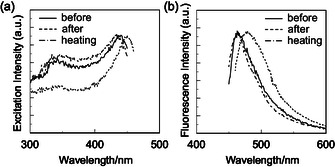
(a) Solid‐state excitation at *λ*_max_^fl‐solid^ and (b) fluorescence spectra (*λ* ^ex^ = *λ*_max_^ex‐solid^) of solid **NI‐3** before and after grinding of as‐recrystallized dye and after heating the ground solid at 150°C. Adapted with permission from ref. [72]. Copyright 2012 Elsevier.

The colors of the as‐recrystallized dyes are light yellow for **NI‐3** and nearly colorless for **NI‐4**. The fluorescence colors under UV lamp (365 nm) are blue for both **NI‐3** and **NI‐4**. The *λ*_max_^ex‐solid^ (441 and 397 nm) and *λ*_max_^fl‐solid^ (463 and 434 nm) of the as‐recrystallized dyes **NI‐3** and **NI‐4** were bathochromically shifted by 69 and 40 nm for **NI‐3** and 22 and 11 nm for **NI‐4**, respectively, compared to those (ICT‐based *λ*_max_^abs‐solution^ and *λ*_max_^fl‐solution^) for the dyes in 1,4‐dioxane (Figure 11). The Φ_fl_‐_solid_ value (0.17) of **NI‐4** was higher than that (0.02) for **NI‐3**, but the Φ_fl_‐_solid_ values of both the dyes were significantly reduced compared to their Φ_fl_‐_solution_ values in 1,4‐dioxane.

The presence or absence of MFC of the carbazole‐based D*–π*–A‐type fluorescent dyes was examined by the following procedure. When the as‐recrystallized dyes **NI‐3** and **NI‐4** were ground at a stress of 50–100 N/cm^2^ in a mortar with a pestle, the *λ*_max_^ex‐solid^ and *λ*_max_^fl‐solid^ for the dyes are bathochromically shifted by 9 and 14 nm for **NI‐3** and 27 and 22 nm for **NI‐4**. However, for both the dyes, the Φ_fl_‐_solid_ values change little by grinding. When the ground samples were heated at 150ºC for **NI‐3** and 110ºC for **NI‐4** (beyond the *T*_c_) or exposed to solvent vapor (e.g., acetone), the colors and fluorescent colors restored to the original ones. The time‐resolved fluorescence measurements of **NI‐3** and **NI‐4** revealed that the fluorescence decay profiles are fitted to biexponential curves with *τ*_1‐solid_ = 0.2–0.4 ns and *τ*_2‐solid_ = 12.2–4.2 ns both before and after grinding. The ratios of fractional contributions (*A*_1_/*A*_2_) of **NI‐3** increased, but that of **NI‐4** decreased after grinding, indicating the existence of two distinct emission states in the dye solids before and after grinding.

**FIGURE 12:**
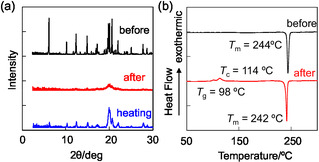
(a) XRD patterns of solid **NI‐3** before (as‐recrystallized) and after grinding of as‐recrystallized dye and after heating the ground solid. (b) DSC curves (heating process with scan rate of 10°C min^**−**1^) of **NI‐3** before and after grinding (*T*_g_: glass transition, *T*_c_: crystallization, and *T*_m_: melting). Adapted with permission from ref. [72]. Copyright 2012 Elsevier.

The XRD measurements of as‐recrystallized dyes **NI‐3** and **NI‐4** showed diffraction peaks assignable to well‐defined microcrystalline structures, whereas the diffraction peaks almost disappeared after grinding, showing that the ground solids were in an amorphous state with reduced molecular orientation and arrangement (Figure 12a). The DSC analysis for of as‐recrystallized dyes **NI‐3** and **NI‐4** indicated that both the dyes showed only one sharp endothermic peak due to melting (*T*_m_) (Figure 12b). After heating the ground solids of both dyes beyond *T*_c_, the XRD patterns of the solids are very similar to those before grinding, suggesting that the microcrystalline structure was restored by heating. On the other hand, the DSC analysis showed the ground solids of **NI‐3** and **NI‐4** underwent an endothermic *T*_g_ at 98°C and 57°C and then an exothermic *T*_c_ at 114°C and 74°C before *T*_m_ at 242°C and 175°C, respectively, indicating the thermal behavior of typical amorphous solids. These results revealed that **NI‐3** and **NI‐4**, which are in the crystalline state before grinding, changed to an amorphous state with a disturbed regularity of molecular orientation and arrangement after grinding.

**FIGURE 13:**
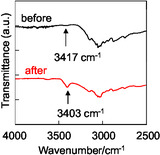
FT**‐**IR spectra of **NI‐3** before and after grinding. Adapted with permission from ref. [72]. Copyright 2012 Elsevier.

**FIGURE 14:**
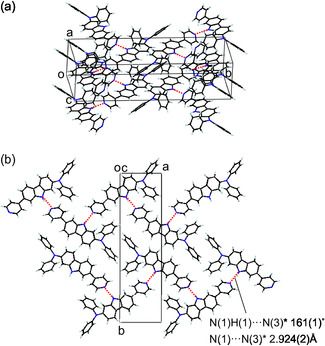
(a) Crystal packing and (b) hydrogen bonding pattern of **NI‐3** (CCDC Deposition Number 810771). Adapted with permission from ref. [72]. Copyright 2012 Elsevier.

Fourier transform infrared spectroscopy (FT‐IR) studies of as‐recrystallized dye **NI‐3** showed that there is no free N—H stretching at the carbazole amino group, whereas the broadened absorption band at 3417 cm^−1^ indicated the formation of a weak hydrogen bond (NH···N) between the proton on the nitrogen atom of the carbazole skeleton and the pyridyl nitrogen atom (Figure 13), which is in full agreement with the single‐crystal X‐ray structure analysis of **NI‐3** (Figure 14). Meanwhile, the N—H stretching of the amino group of the carbazole skeleton at 3403 cm^−1^ which is observed in the FT‐IR spectrum of the ground solids (amorphous state), becomes sharper than that observed in the as‐recrystallized dye, indicating the formation of stronger intermolecular hydrogen bonds.

Single‐crystal X‐ray structural analysis of **NI‐3** demonstrated that the dye molecules are linked by intermolecular NH···N hydrogen bonds between the protons on the nitrogen atom of carbazole skeleton and the pyridyl nitrogen atom to form one‐dimensional molecular chains: the protons on the nitrogen atom of carbazole skeleton in one dye molecule points toward the pyridyl nitrogen atom of the neighboring dye molecule (N(1)H(1)···N(3)*angle; 161(1)°, N(1)···N(3)*distance; 2.924(2)Å) (Figure 14). Furthermore, no formation of *π* *–π* interactions between the dye molecules was observed. This result suggests that the formation of continuous intermolecular hydrogen bond between the dye molecules is a main factor causing a remarkable bathochromic shifts of *λ*_max_^abs^ and *λ*_max_^fl^ and a significant solid‐state fluorescence quenching with changing from the solution to the crystalline state [94]. Although single crystals of **NI‐4** were not large enough to perform X‐ray structural analysis, the relatively high Φ_fl_‐_solid_ value for the as‐recrystallized dye **NI‐4** indicates that the *N*‐butylation of carbazole skeleton can effectively prevent the formation of continuous intermolecular hydrogen bond between the dye molecules. On the other hand, it was suggested that the formation of intermolecular *π* *–π* interactions between the dye molecules causes the bathochromic shift of *λ*_max_^abs^ and *λ*_max_^fl^ and the lowering of Φ_fl_ for **NI‐4** from the solution to the crystalline state.

Based on the above experimental results and theoretical MO calculations, the b‐MFC of the carbazole‐based D*–π*–A‐type fluorescent dyes **NI‐3** and **NI‐4** was discussed. The bathochromic shifts of *λ*_max_^abs^ and *λ*_max_^fl^ and the lowering of Φ_fl_ with changing from the solution to the crystalline state are attributed to a continuous intermolecular hydrogen bonds for **NI‐3** and the intermolecular *π* *–π* interactions for **NI‐4** in the crystalline state. Moreover, for the dye **NI‐3**, the intermolecular hydrogen bonds in the ground solids (amorphous state) become stronger than that in the as‐recrystallized dye. Consequently, it may be concluded that the b‐MFC of **NI‐3** and **NI‐4** are caused by the formations of the stronger hydrogen bond for **NI‐3** and the stronger intermolecular *π* *–π* interactions for **NI‐4** upon transition from the crystalline state to the amorphous state (Figure 9). On the other hand, for the carbazole‐based D*–π*–A‐type fluorescent dyes, the Φ_fl_‐_solid_ value hardly changes from the crystalline to the amorphous state. This result indicates that compared to the crystalline state with long‐range order in molecular orientation and arrangement (long‐range intermolecular interactions), a nonradiative decay pathway of the excited states is relatively inhibited in the amorphous states with the short‐range hydrogen bonds and the *π* *–π* interactions, although the intermolecular interactions in the amorphous state became stronger than those in the crystalline state. Meanwhile, we demonstrated that the heteropolycyclic D*–π*–A‐type fluorescent dye with a dipole moment of ca. 5 Debye exhibit the pronounced b‐MFC due to the change in the dipole–dipole interactions between the crystalline state and the amorphous state (Figure 3). Therefore, the relatively weak b‐MFC for **NI‐3** and **NI‐4** is likely to their small dipole moments (*μ*_g_ = ca. 3.6 Debye).

Furthermore, to clarify the effect of intermolecular interactions between dye molecules on the MFC of carbazole‐based D*–π*–A‐type fluorescent dyes, we designed and synthesized **SO1** and **SO2**, which have a nitro group as a strong electron‐withdrawing substituent (Figure 10) [73]. **SO2** also has *n*‐butyl group on the nitrogen atom of the carbazole skeleton.

**FIGURE 15:**
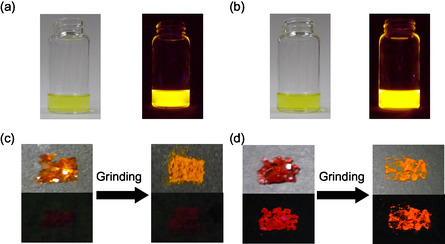
Photographs of (a) **SO1** and (b) **SO2** in 1,4‐dioxane under room light (left) and under UV‐light irradiation (right). Photographs of powder of (c) **SO1** and (d) **SO2** under room light (top) and under UV irradiation (down) before and after grinding. Adapted with permission from ref. [73]. Copyright 2012 John Wiley & Sons, Inc.

The 1,4‐dioxane solutions of **SO1** and **SO2** showed an photoabsorption band at around 310 nm (*ε*_max_ = 38 000 and 31 600 M^–1 ^cm^–1^, respectively) due to the *π* → π* transition and an photoabsorption band at around 399 nm (*ε*_max_ = 31 900 and 26 000 M^–1 ^cm^–1^, respectively) due to the strong ICT excitation characteristics from the diphenylamino group to the *p*‐nitrophenyl group. The *λ*_max_^fl‐solution^ of both the dyes appeared at around 580 nm. In addition, the Φ_fl_‐_solution_ values of **SO1** and **SO2** were 0.32 and 0.35, respectively. Therefore, it was found that the ICT‐based *λ*_max_^abs‐solution^ and *λ*_max_^fl‐solution^ of **SO1** and **SO2** were bathochromically shifted and their Φ_fl_‐_solution_ values were lowered, compared to those of **NI‐3** and **NI‐4** with pyridine ring as a moderate electron‐withdrawing group. The solution color of both the dyes in 1,4‐dioxane was yellow, and their fluorescence color under UV lamp (365 nm) was yellow–orange. These results indicate that, as with **NI‐3** and **NI‐4**, the *N‐*alkylation of the carbazole skeleton has only a small effect on the optical properties of the dyes (Figure 15a,b).

The MO calculations indicated that for **SO1** and **SO2,** the HOMO is distributed over the carbazole skeleton containing the diphenylamino group and the LUMO is mainly localized on the *p*‐nitrophenyl group (Figure 5). Furthermore, the change in charge density accompanying the first electron transition suggested that both the dyes exhibit strong ICT characteristics from the diphenylamino–carbazole moiety to *p*‐nitrophenyl group upon photoexcitation. The dipole moments (*μ*_g_) of **SO1** and **SO2** in the ground state were ca. 7.6 Debye, which is much larger than those of **NI‐3** and **NI‐4** (ca. 3.6 Debye).

**FIGURE 16:**
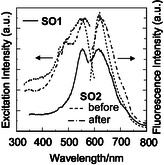
Solid‐state excitation at *λ*_max_^fl‐solid^ and fluorescence spectra (*λ* ^ex^ = *λ*_max_^ex‐solid^) of **SO1** and **SO2** before and after grinding. Adapted with permission from ref. [73]. Copyright 2012 John Wiley & Sons, Inc.

The colors of as‐recrystallized dyes **SO1** and **SO2** were orange and red, respectively (Figure 15c,d). Interestingly, under UV lamp (365 nm), the as‐recrystallized dye **SO2** exhibited red fluorescence emission, although the as‐recrystallized dye **SO1** did not show any observable. The *λ*_max_^ex‐solid^ and *λ*_max_^fl‐solid^ of **SO1** (557 and 618 nm) and **SO2** (549 and 618 nm) are bathochromically shifted by 158 and 39 and 150 and 39 nm, respectively, compared with those in 1,4‐dioxane (Figure 16). The Φ_fl_‐_solid_ value of **SO2** in the solid state is 0.03, which is smaller than that in 1,4‐dioxane. It was difficult to accurately evaluate the Φ_fl_‐_solid_ value of **SO1**, because the fluorescence of **SO1** is strongly quenched in the solid state. Meanwhile, by grinding the as‐recrystallized dyes **SO1** and **SO2** in a mortar with a pestle, the *λ*_max_^ex‐solid^ and *λ*_max_^fl‐solid^ for **SO2** were bathochromically shifted by 15 and 7 nm, respectively. However, no change in the Φ_fl_‐_solid_ value was observed by grinding. In contrast to the dye **SO2**, the dye **SO1** showed no significant changes in the *λ*_max_^ex‐solid^ and *λ*_max_^fl‐solid^ upon grinding.

**FIGURE 17:**
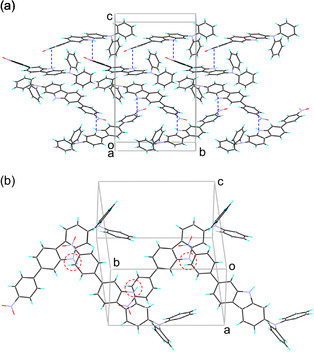
(a) Crystal packing and (b) a continuous intermolecular *π*–stacking of **SO1** (CCDC Deposition Number 878842). Dotted lines and circles show the nonbonded overlapping atoms between the neighboring molecules. Adapted with permission from ref. [73]. Copyright 2012 John Wiley & Sons, Inc.

**FIGURE 18:**
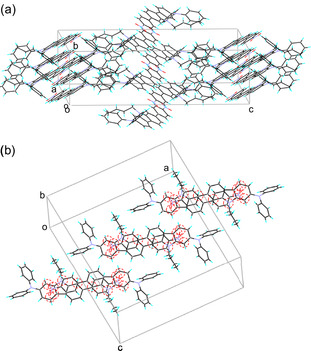
(a) Crystal packing and (b) dimer unit of **SO2** (CCDC Deposition Number 878843). Dotted circles show the short interatomic contacts of less than 3.6 Å in a pair of dye molecules. Adapted with permission from ref. [73]. Copyright 2012 John Wiley & Sons, Inc.

In order to elucidate the effects of the molecular packing structure on the solid‐state fluorescence properties, the X‐ray crystal structures of **SO1** and **SO2** have been determined. A continuous intermolecular *π*‐stacking among the dye molecules was observed in the crystal structure of **SO1** in which partial *π*‐overlapping was formed between the *p*‐nitrophenyl group and the carbazole moiety of the neighboring molecules (Figure 17). The shortest distance of nonbonded overlapping atoms is 3.60 Å between the nitrogen atom of the carbazole ring and the carbon atom of the *p*‐nitrophenyl group. On the other hand, the crystal structure of **SO2** is constructed by a dimer unit which is composed of a pair of dye molecules (Figure 18). The pair of dye molecules has 12 short interatomic contacts of less than 3.6 Å, and the *π*‐overlappings were observed over the whole molecule from the nitrophenyl group to carbazole moiety. The interplanar distances are ca. 3.54 Å, which suggests the formation of intermolecular *π* *–π* interactions between the dye molecules in the dimer unit.

Therefore, the X‐ray structural analysis revealed that the formation of a continuous intermolecular *π*‐stacking or intermolecular *π* *–π* interactions is responsible for a large bathochromic shift of the *λ*_max_^abs^ and *λ*_max_^fl^ and a decrease in the Φ_fl_ values for **SO1** and **SO2** from the solution to the crystalline state. However, the Φ_fl_‐_solid_ value (∼0) of **SO1** is significantly lower than that of **SO2** (Φ_fl_‐_solid_ = 0.03). It was concluded that compared with the formation of strong intermolecular *π* *–π* interactions composed of a pair of dye molecules in the crystal structure of **SO2**, the continuous intermolecular *π*‐stacking among the dye molecules in the crystal structure of **SO1** induces the larger delocalization or deactivation of excitons, leading to the drastic solid‐state fluorescence quenching [91–93]. These results also show that the introduction of bulky substituent to the carbazole ring of the D*–π*–A fluorescent dyes can effectively prevent the formation of continuous intermolecular *π*‐stacking among fluorophores.

As with the heteropolycyclic D*–π*–A‐type fluorescent dyes with a *μ*_g_ value of ca. 5 Debye, the XRD measurements for the recrystallized dye **SO2** showed diffraction peaks due to a well‐defined microcrystalline structure, whereas after grinding, these peaks almost disappeared, indicating that the crystal lattice was significantly disordered. Thus, it was suggested that the weak b‐MFC for **SO2** is ascribable to the large *μ*_g_ value (ca. 8.0 Debye), that is, the strong dipole–dipole interaction between the D*–π*–A‐type fluorescent dye molecules may inhibit the change in molecular arrangement between crystalline and amorphous states by the grinding, preventing the expression of MFC. In addition, non‐MFC for **SO1** may be due to strong continuous intermolecular *π*‐stacking that prevents the destruction of the molecular arrangement by grinding.

**FIGURE 19:**
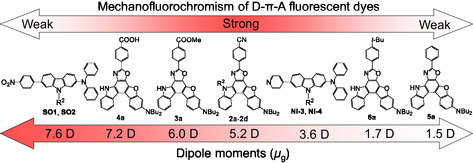
Correlation between the b‐MFC and the dipole moment of D*–π*–A‐type fluorescent dyes. The degrees of MFC were evaluated as the value of differences (Δ*λ*_max_^fl‐solid^) in *λ*_max_^ex‐solid^ before and after grinding. Adapted with permission from ref. [74]. Copyright 2022 Royal Society of Chemistry.

Consequently, our work on MFC of D*–π*–A‐type fluorescent dyes revealed that the b‐MFC of the D*–π*–A‐type fluorescent dye is due to the reversible changes in not only intermolecular *π* *–π* interactions and dipole–dipole interactions but also to the formation of intermolecular hydrogen bonds between the crystalline state before grinding and the amorphous state after grinding. Furthermore, we proposed that the most important point for expressing the pronounced b‐MFC is to construct a D*–π*–A‐type fluorescent dye structure with a moderate dipole moment (ca. 5 Debye), which can be precisely controlled by adjusting the electron‐donating ability of D moiety, the electron‐accepting ability of A moiety, the steric size of the substituents, and the magnitude of donor–acceptor *π*‐conjugated system (Figure 19).

## 5 MFC of Carbazole‐Based (D*–π*–)_2_A‐Type Fluorescent Dyes

**FIGURE 20:**
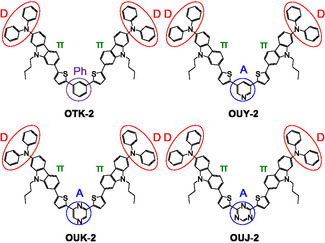
Chemical structures of (D*–π*–)_2_Ph‐type fluorescent dye **OTK‐2** and (D*–π*–)_2_A‐type fluorescent dyes **OUY‐2**, **OUK‐2**, and **OUJ‐2**.

In order to further gain insight into the molecular design directions of D*–π*–A‐type mechnofluorochromic dyes, we designed and developed (D*–π*–)_2_A‐type fluorescent dyes **OUY‐2**, **OUK‐2**, and **OUJ‐2**, which consist of two diphenylamino groups as the D moiety, a pyridine, pyrazine, or triazine ring as the A unit, and two carbazole–thiophene skeletons as *π*‐conjugated system, and a (D*–π*–)_2_Ph‐type fluorescent dye **OTK‐2**, which has a phenyl (Ph) group instead of an azine ring (Figure 20) [74]. Compared with D*–π*–A‐type fluorescent dyes, (D*–π*–)_2_A‐type fluorescent dyes show higher molar extinction coefficients and fluorescence quantum yields due to their strong ICT characteristics and are therefore expected to show an obvious MFCs, i.e., high fluorescent color contrasts, before and after grinding. In fact, the (D*–π*–)_2_A‐type fluorescent dyes were found to show h‐MFC or b‐MFC, as described below.

The toluene solutions of **OUY‐2**, **OUK‐2**, and **OUJ‐2** show the *λ*_max_^abs‐solution^ at 398, 401, and 433 nm, respectively, which are attributed to the ICT excitation characteristics from the two carbazole–thiophene skeletons containing two diphenylamino groups (the D–*π* moieties) to the pyridine, pyrazine or triazine ring (the A unit). A shoulder band was observed at ca. 430 nm for **OUK‐2**. Indeed, the ICT‐based photoabsorption bands appear in the longer wavelength region in the order **OUY‐2** < **OUK‐2** < **OUJ‐2**, which is consistent with the increasing electron‐withdrawing ability in the order pyridyl < pyrazyl < triazyl group. The *ε*_max_ values for the ICT‐based *λ*_max_^abs‐solution^ of **OUY‐2**, **OUK‐2**, and **OUJ‐2** are ca. 100 000, 75 000, and 80 000 M^−1^ cm^−1^, respectively. The *λ*_max_^fl‐solution^ appear at a longer wavelength region in the order of **OUY‐2** (453 nm) < **OUK‐2** (480 nm) < **OUJ‐2** (509 nm). The Φ_fl_‐_solution_ value become higher in the order of **OUY‐2** (0.38) < **OUK‐2** (0.48) < **OUJ‐2** (0.81). Thus, it was found that the *λ*_max_^abs‐solution^ and *λ*_max_^fl‐solution^ of **OUY‐2** appear at a longer wavelength region by ca. 25 nm, compared to that (375 and 429 nm) of the corresponding D*–π*–A‐type fluorescent **NI‐4**. The Φ_fl_‐_solution_ value of **OUY‐2** is lower than that (ca. 0.8) of **NI‐4**, although the *ε*_max_ for the ICT‐based *λ*_max_^abs‐solution^ of **OUY‐2** is much higher than those (33 000 M^−1^ cm^−^) of **NI‐4**. The ICT‐based *λ*_max_^abs‐solution^ (395 nm) and *λ*_max_^fl‐solution^ (447 nm) of **OTK‐2** appeared at a shorter wavelength region compared to those of **OUY‐2**, **OUK‐2**, and **OUJ‐2**, although the *ε*_max_ for **OTK‐2** is comparable to that of the three dyes. The *τ*_f_‐_solution_ value increases in the following order: **OTK‐2** (0.62 ns) < **OUY‐2** (0.82 ns) < **OUK‐2** (1.60 ns) < **OUJ‐2** (1.92 ns). In toluene solution, **OTK‐2** and **OUY‐2** were almost colorless, **OUK‐2** and **OUJ‐2** were greenish yellow. The fluorescent colors were blue for **OTK‐2** and **OUY‐2**, light blue for **OUK‐2**, and green for **OUJ‐2**.

**FIGURE 21:**
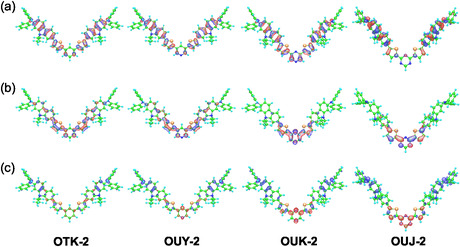
(a) HOMO and (b) LUMO of **OTK‐2**, **OUY‐2**, **OUK‐2**, and **OUJ‐2** derived from MO calculations. The blue and red lobes represent the negative and positive signs of the MO coefficients. The magnitude of each lobe corresponds to the MO coefficient. (c) Calculated electron density changes accompanying the first electronic excitation of **OTK‐2**, **OUY‐2**, **OUK‐2**, and **OUJ‐2**. The red and blue lobes represent the increase and decrease in electron density accompanying the electronic transition, respectively. The area of the lobe indicates the magnitude of the electron density change. Adapted with permission from ref. [74]. Copyright 2022 Royal Society of Chemistry.

The MO calculations revealed that the HOMOs of **OUY‐2, OUK‐2**, and **OUJ‐2** are distributed over the two carbazole‐thiophene skeletons containing two diphenylamino groups. Meanwhile, the LUMOs for of **OUY‐2, OUK‐2**, and **OUJ‐2** are mainly localized on the thienylpyridine moiety, the thienylpyrazine moiety, and the thienyltriazine moiety, respectively. The HOMO and LUMO of **OTK‐2** are distributed throughout the dye molecular structure (Figure 21). The changes in the electron density upon the first electron excitation suggested that the four dyes exhibit the ICT characteristics from the two carbazole–thiophene skeletons containing two diphenylamino groups (the D–*π* moieties) to each azine ring (the A unit) or Ph group. Notably, the magnitude of change in the electron density of **OUJ‐2** is larger than those of **OUY‐2** and **OUK‐2**, so that **OUJ‐2** has strong ICT characteristics. Moreover, it was found that the *μ*_g_ value is 4.0 Debye for **OTK‐2**, 1.4 Debye for **OUY‐2**, 3.2 Debye for **OUK‐2**, and 2.9 Debye for **OUJ‐2**.

**FIGURE 22:**
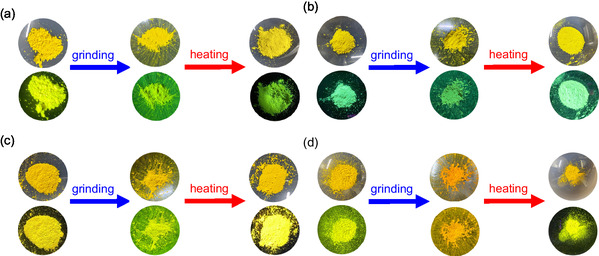
Photographs of powder of (a) **OTK‐2**, (b) **OUY‐2**, (c) **OUK‐2**, and (d) **OUJ‐2** under room light (top) and under UV‐light irradiation (down) before and after grinding of as‐recrystallized dye and after heating the ground solids. Adapted with permission from ref. [74]. Copyright 2022 Royal Society of Chemistry.

**FIGURE 23:**
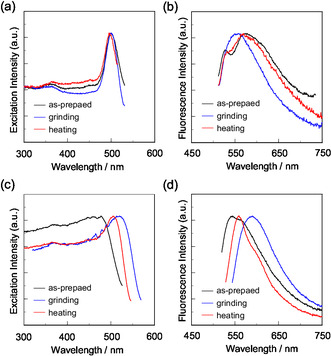
(a) Solid‐state excitation at *λ*_max_^fl‐solid^ and (b) fluorescence spectra (*λ* ^ex^ = *λ*_max_^ex‐solid^) of solid **OUK‐2** before and after grinding of as‐recrystallized dye and after heating the ground solid at 180°C. (c) Solid‐state excitation at *λ*_max_^fl‐solid^ and (d) fluorescence spectra (*λ* ^ex^ = *λ*_max_^ex‐solid^) of solid **OUJ‐2** before and after grinding of as‐recrystallized dye and after heating the ground solid at 200°C. Adapted with permission from ref. [74]. Copyright 2022 Royal Society of Chemistry.

The colors of the as‐recrystallized dyes are yellow for **OUK‐2** and **OUJ‐2** and yellowish orange for **OTK‐2** and **OUY‐2**. The fluorescent colors under UV lamp (365 nm) are yellow for **OUK‐2**, light green for **OUY‐2**, and greenish yellow for **OTK‐2** and **OUJ‐2** (Figure 22). The *λ*_max_^ex‐solid^ and *λ*_max_^fl‐solid^ of the as‐recrystallized dyes appeared at 484 and 543 nm for **OTK‐2**, 461 and 501 nm for **OUY‐2**, 501 and 575 nm for **OUK‐2**, and 477 and 545 nm for **OUJ‐2**, which exhibited significant bathochromically shift by 89 and 96, 63 and 48, 100 and 95, and 44 and 36 nm, respectively, compared to those in the toluene solution (Figure 23). Notably, similar to the solution state, the as‐recrystallized dyes **OTK‐2**, **OUY‐2**, and **OUK‐2** showed a vibronically structured fluorescence band, but **OUK‐2** and **OUJ‐2** exhibited a broadened fluorescence band with shoulder band at 527 and 562 nm, respectively. The Φ_fl‐solid_ values of the as‐recrystallized dyes **OTK‐2**, **OUY‐2**, **OUK‐2**, and **OUJ‐2** are 0.15, 0.08, 0.04, and 0.12, which are significantly lower than those in the solution.

When the as‐recrystallized dyes were ground, the color and fluorescence of **OUJ‐2** changed to orange (Figure 22). The fluorescent color of **OUK‐2** changed to greenish yellow upon grinding, but the color remained unchanged. However, the as‐recrystallized **OTK‐2** and **OUY‐2** showed no obvious changes in the color and the fluorescent color upon grinding. For the as‐recrystallized **OTK‐2**, no significant changes in the *λ*_max_^ex‐solid^ and *λ*_max_^fl‐solid^ were observed upon grinding. The *λ*_max_^fl‐solid^ of **OUY‐2** and **OUK‐2** were bathochromically shifted and hypsochromically shifted, respectively, while the *λ*_max_^ex‐solid^ showed little change by grinding (Figure 23a,b). Therefore, these results indicated that **OUY‐2** exhibited nonobvious b‐MFC, whereas **OUK‐2** exhibited h‐MFC. The vibrational structure of the fluorescence bands disappeared after grinding in **OTK‐2**, **OUY‐2**, and **OUK‐2**. However, **OUJ‐2** showed a significant bathochromic shift of the *λ*_max_^ex‐solid^ and *λ*_max_^fl‐solid^ by grinding (Figure 23c,d), that is, a pronounced b‐MFC, compared with **OUY‐2**. The degrees of MFC, which were evaluated as the absolute value of differences (Δ*λ*_max_^ex‐solid^ and Δ*λ*_max_^fl‐solid^) in *λ*_max_^ex‐solid^ and *λ*_max_^fl‐solid^ before and after grinding, increased in the following order: **OTK‐2** (0 and −5 nm) < **OUY‐2** (+2 and + 7 nm) < **OUK‐2** (0 and −17 nm) ≪ **OUJ‐2** (+43 and +45 nm). It is noteworthy that the Δ*λ*_max_^fl‐solid^ values between the shoulder fluorescence band (*λ*_max_^shfl‐solid^) of the as‐recrystallized dyes and the *λ*_max_^fl‐solid^ of the solids after grinding are +31 nm for **OUK‐2** and +28 nm for **OUJ‐2**, suggesting that **OUK‐2** and **OUJ‐2** exhibit the b‐MFC. The Φ_fl‐solid_ values of **OTK‐2**, **OUY‐2**, **OUK‐2**, and **OUJ‐2** increased slightly with grinding from 0.15, 0.08, 0.04, and 0.12 to 0.16, 0.10, 0.06, and 0.17, respectively. Furthermore, the *τ*_fl‐solid_ value of as‐recrystallized **OTK‐2** was shortened from 0.93 to 0.73 ns before and after grinding, but those of **OUY‐2**, **OUK‐2**, and **OUJ‐2** were lengthened from 1.02, 2.04, and 2.14 ns to 1.05, 2.16, and 5.29 ns before and after grinding, respectively. The *τ*_fl‐solid_ value of **OUJ‐2** increased 2.5 times after grinding compared to before grinding. By heating of the ground solids at 180–200°C (beyond the *T*_c_) or exposure of it to solvent vapor (e.g., THF) for several minutes, the *λ*_max_^ex‐solid^ and *λ*_max_^fl‐solid^ as well as the color and fluorescent color of **OTK‐2** and **OUK‐2** recovered to the original ones before grinding, but those of **OUY‐2** and **OUJ‐2** were different from those before grinding.

**FIGURE 24:**
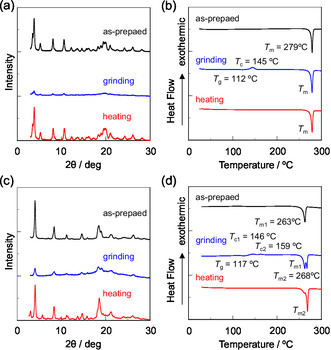
XRD patterns of (a) **OUK‐2** and (b) **OUJ‐2** and DSC curves (heating process with scan rate of 10°C min^**−**1^) of (c) **OUK‐2** and (d) **OUJ‐2** before and after grinding of as‐recrystallized dye and after heating the ground solid (*T*_g_: glass transition, *T*_c_: crystallization, and *T*_m_: melting). Adapted with permission from ref. [74]. Copyright 2022 Royal Society of Chemistry.

The XRD patterns of the as‐recrystallized dyes **OTK‐2**, **OUY‐2**, **OUK‐2**, and **OUJ‐2** can be assigned to diffraction patterns due to well‐defined microcrystalline structures (Figure 24a,c). The diffraction peaks almost fully disappeared after grinding, indicating significant disruption of the crystal lattice. On the other hand, the diffraction patterns of **OTK‐2** and **OUK‐2** after heating the ground solids are similar to those of the as‐recrystallized dyes before grinding, indicating that the microcrystalline structure was restored by heating. Furthermore, the DSC analysis revealed that the as‐recrystallized dyes **OUK‐2** and **OUJ‐2** only exhibited sharp endothermic peak due to *T*_m_ at 279°C and 263°C, respectively (Figure 24b,d). However, the as‐recrystallized dyes **OTK‐2** and **OUY‐2** showed typical DSC traces attributable to polymorphic mixtures. The as‐recrystallized dye **OTK‐2** exhibited a *T*_m1_ at 249°C, followed by a *T*_c_ at 258°C, and then melted at 272°C (*T*_m2_). It is worth mentioning here that **OUY‐2** has three crystal forms, that is, crystal polymorphism. Indeed, the DSC traces of the as‐recrystallized dye **OUY‐2** exhibited a *T*_m1_ at 230°C, *T*_c1_ at 233°C, *T*_m2_ at 262°C, *T*_c2_ at 269°C, and then *T*_m3_ at 278°C. The MFC based on crystal polymorphism is also of scientific interest. In fact, Liu et al. found that benzimidazolylaryl acrylonitrile derivative crystallizes in two polymorphs; one is a sheet‐like crystal (OR‐phase crystal) emitting orange–red fluorescence and the other one is a block‐like crystal (G‐phase crystal) showing green fluorescence. They revealed that the OR‐phase crystal showed remarkable h‐OHC, but the G‐phase crystal are not mechnofluorochromic [46]. The DSC traces of the ground solids of **OTK‐2**, **OUY‐2**, **OUK‐2**, and **OUJ‐2** are typical of amorphous solids: the ground solids showed an endothermic *T*_g_, an exothermic *T*_c_, and then *T*_m_. It is noteworthy here that when the ground solids in the amorphous state were heated, **OUY‐2** did not restore to its original microcrystals but transformed to a more stable microcrystals exhibiting *T*_m3_ at 278°C, while **OUJ‐2** transformed to another microcrystalline structure exhibiting *T*_m2_ at 268°C, which is different from the DSC trace of the as‐recrystallized microcrystals. The solids obtained after heating the ground solids at 180°C–200°C beyond the *T*_c_ showed the DSC traces with only *T*_m_ for **OUY‐2**, **OUK‐2**, and **OUJ‐2**, whereas the DSC traces of **OTK‐2** showed one *T*_c_ and two *T*_m_ due to its crystal polymorphism. Therefore, the XRD and DSC measurements demonstrated that when a ground solid in the amorphous state is heated, it will either recrystallize to restore its original microcrystals or undergo a polymorphic transformation to form other microcrystals. The Archimedean method demonstrated that the density of the solids for **OTK‐2**, **OUY‐2**, **OUK‐2**, and **OUJ‐2** increased upon grinding from 1.40, 1.26, 1.25, and 1.13 g cm^−3^ to 1.45, 1.37, 1.39, and 1.31 g cm^−3^, respectively. This indicates that the dye molecules were more densely packed in the amorphous state after grinding, compared to the crystalline state before grinding.

Based on the above experimental and theoretical MO calculation results, the mechanism for MFC of (D*–π*–)_2_A‐type fluorescent dyes is discussed below. Similar to the case of D*–π*–A‐type fluorescent dyes described in Sections 3 and 4, in the crystalline state of (D*–π*–)_2_A‐type fluorescent dyes, the dye molecules are stacked due to long‐range intermolecular *π* *–π* interactions between the neighboring dye molecules, and the dipole moments are oriented antiparallel to maximize the intermolecular dipole–dipole interactions, resulting in the bathochromic shifts of *λ*_max_^abs^ and *λ*_max_^fl^ upon transition from the solution to the crystalline state. Furthermore, the delocalization of excitons due to the formation of long‐range intermolecular *π* *–π* interactions in the crystalline state leads to a nonradiative decay pathway of the excited state, resulting in a decrease in the Φ_fl_ value in the crystalline state [91–93]. Grinding the as‐recrystallized microcrystalline dyes brings the dye molecules closer together, maximizing the dipole–dipole interactions and the intermolecular *π* *–π* interactions, which is evidenced by the increased density of the dye molecules in the amorphous state. Dye molecules with large dipole moments have the increased dipole–dipole interaction strength. Indeed, the *µ*_g_ values (3.2 Debye and 2.9 Debye) of **OUK‐2** and **OUJ‐2** are larger than that (1.4 Debye) of **OUY‐2**, and thus, **OUK‐2** and **OUJ‐2** have the (D*–π*–)_2_A structure with moderate dipole moment due to relatively strong electron‐withdrawing pyrazyl or triazyl group as the A unit. Consequently, the pronounced b‐MFC of **OUJ‐2** can be attributed to the reversible switching between the crystalline state and amorphous state, accompanied by changes in dipole–dipole interaction between the dye molecules due to its moderate dipole moment. On the other hand, the weak b‐MFC of **OUY‐2** may be attributed to the small change in dipole–dipole interactions between dye molecules upon transition from the crystalline state to the amorphous state due to its small dipole moment (*μ*_g_ = 1.4 Debye), which is in common with nonobvious b‐MFC of D*–π*–A‐type fluorescent dyes **4a** and **5a**, which also have small dipole moments (*μ*_g_ = ca. 1−2 Debye) (Figure 19) [69–71]. Meanwhile, we speculate that the moderate ICT characteristics of **OUK‐2** may induce not only apparent b‐MFC attributed to strong dipole–dipole interactions but also h‐MFC attributed to the distortion and twist between the D*–π* moiety and the A unit. On the other hand, the Φ_fl‐solid_ value in the amorphous state is slightly higher than that in the crystalline state, suggesting that, in contrast to the long‐range intermolecular *π* *–π* interactions between the dye molecules in the crystalline state, the short‐range intermolecular *π* *–π* interactions between the dye molecules in the amorphous state suppress the delocalization of excitons. Furthermore, considering the fact that the (D*–π*–)_2_Ph structure (**OTK‐2**) with a relatively strong dipole moment (*μ*_g_ = 4.0 Debye) but without any electron‐withdrawing group showed insignificant MFC, it was suggested that the (D*–π*–)_2_A structure with strong ICT characteristics is essential to exhibit pronounced MFC.

In this work, we demonstrated that carbazole‐based (D*–π*–)_2_A‐type fluorescent dyes with a azine ring as the A unit exhibit h‐MFC or b‐MFC. The experimental and theoretical MO calculation results showed that the MFC of the (D*–π*–)_2_A‐type fluorescent dyes is due to the reversible switching of intermolecular dipole–dipole and *π* *–π* interactions between the as‐recrystallized dye in the crystalline state and the ground dye in the amorphous state. Furthermore, this work revealed that carbazole‐based (D*–π*–)_2_A‐type fluorescent dyes with moderate dipole moments (ca. 3 Debye) and ICT characteristics are capable of activating MFC.

## 6 MFC of D–*π*–A‐Type Pyridinium Fluorescent Dyes

**FIGURE 25:**
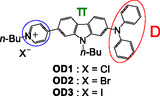
Chemical structures of D*–π*–A‐type pyridinium dyes **OD1**–**3**.

We found that D*–π*–A‐type pyridinium fluorescent dyes **OD1**–**3**, which have a diphenylamino group as D moiety and a pyridinium ring as A moiety connected by carbazole skeleton as *π*‐conjugated bridge, and bearing various counter anions (X^–^ = Cl^–^, Br^–^, or I^–^), exhibit a pronounced b‐MFC. The degrees of b‐MFC depended on the type of counter anion of **OD1**–**3** (Figure 25) [75].

The dyes **OD1**–**3** in 1,4‐dioxane show *λ*_max_^abs‐solution^ at around 440 nm, which is assigned to ICT excitation characteristics from diphenylamino group (D) to pyridinium ring (A), and the corresponding *λ*_max_^fl‐solution^ occurred at around 590 nm. The Φ_fl_‐_solution_ values decreased in the order of **OD1** (0.27) > **OD2** (0.16) > **OD3** (0.02) due to the heavy atom effect of halide ion, that is, the formation of a triplet state via an intersystem crossing (S1 → T1). For the three dyes, the changes in the electron density upon the first electron excitations by the MO calculations showed a strong ICT characteristics from the diphenylamino–carbazole moiety to the pyridinium moiety (Figure 5). The *μ*_g_ values of **OD1**–**3** are ca. 20 Debye, which is much larger than that of typical neutral D*–π*–A‐type fluorescent dyes.

**FIGURE 26:**
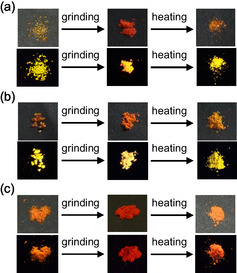
Photographs of powder of (a) **OD‐1**, (b) **OD‐2**, and (c) **OD‐3** under room light (top) and under UV‐light irradiation (down) before and after grinding of as‐recrystallized dye and after heating the ground solids. Adapted with permission from ref. [75]. Copyright 2013 Elsevier.

**FIGURE 27:**
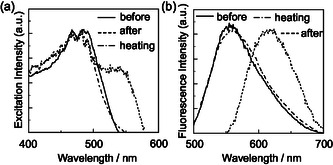
(a) Solid‐state excitation at *λ*_max_^fl‐solid^ and (b) fluorescence spectra (*λ* ^ex^ = *λ*_max_^ex‐solid^) of solid **OD‐2** before and after grinding of as‐recrystallized dye and after heating the ground solid at 170°C. Adapted with permission from ref. [75]. Copyright 2013 Elsevier.

For the as‐recrystallized dyes, the colors are yellowish orange for **OD1** and **OD2** and orange for **OD3**. The corresponding fluorescence colors are yellow for **OD1** and **OD2** and orange for **OD3** under UV lamp (365 nm) (Figure 26). The *λ*_max_^ex‐solid^ of **OD1**–**3** appeared at 506, 482, and 519 nm, respectively, which were bathochromically shifted by 64, 40, and 74 nm, respectively, compared with the *λ*_max_^abs‐solution^ in 1,4‐dioxane (Figure 27). The corresponding *λ*_max_^fl‐solid^ of **OD1**–**3** appeared at 552, 553, and 582 nm, respectively, which was hypsochromically shifted by 37, 36, and 3 nm, respectively, compared with the *λ*_max_^fl‐solution^ in 1,4‐dioxane. The Φ_fl_‐_solid_ values of **OD1**–**3** were 0.11, 0.05, and 0.06, respectively, which were lower for **OD1** and **OD2** than the Φ_fl_‐_solution_ values in 1,4‐dioxane.

As a result of investigating the MFC of **OD1**–**3**, the color of as‐recrystallized dye **OD2** changed from yellowish orange to orange, and the fluorescence color changed from yellow to yellowish orange by grinding (Figure 26b). On the other hand, the as‐recrystallized dyes **OD1** and **OD3** showed the change from yellowish orange and orange to red–orange, respectively, and the corresponding fluorescence color showed the change from yellow to orange and orange to red, respectively, by grinding (Figure 26a,c). After grinding, the *λ*_max_^ex‐solid^ and *λ*_max_^fl‐solid^ for **OD1**–**3** are bathochromically shifted by 52 and 69, 48 and 60, and 59 and 52 nm, respectively (Figure 27). The Φ_fl_‐_solid_ values (0.02–0.06) for the three dyes decreased by grinding. When the ground solids of **OD2** and **OD3** were heated at ca. 170ºC (beyond *T*_c_), the colors and fluorescent colors almost completely restored to their original state. The fact indicates that the for D*–π*–A‐type pyridinium fluorescent dyes, the MFC, that is, the change in fluorescence color by grinding can be favorably adjusted by changing the type of counter anion. Furthermore, it is noteworthy that the D*–π*–A‐type pyridinium fluorescent dye **OD1**–**3**, which has a considerably large dipole moment (ca. 20 Debye), exhibits a remarkable b‐MFC, despite our work on the b‐MFC of neutral D*–π*–A‐type fluorescent dyes suggested that the D*–π*–A‐type fluorescent dye structures with dipole moments of less than ca. 1–2 Debye or more than ca.8 Debye exhibit weak b‐MFC [69–73].

**FIGURE 28:**
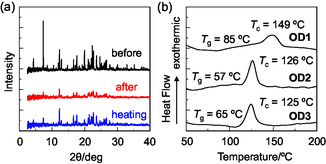
(a) XRD patterns of solid **OD‐2** before and after grinding of as‐recrystallized dye and after heating the ground solid. (b) DSC curves (heating process with scan rate of 10°C min^**−**1^) of **OD1**–**3** after grinding (*T*_g_: glass transition and *T*_c_: crystallization). Adapted with permission from ref. [75]. Copyright 2013 Elsevier.

The XRD measurements with the as‐recrystallized dyes **OD1**–**3** showed diffraction patterns attributed to well‐defined microcrystalline structures (Figure 28a). The diffraction peaks almost disappeared after grinding, indicating that the ground solids were in an amorphous state with reduced molecular orientation and arrangement. On the other hand, it was found from thermogravimetry‐differential thermal analysis analysis that the as‐recrystallized dyes **OD1**–**3** showed decomposition at 250°C–280°C without melting. The DSC analysis for the three dyes indicated that the ground solids **OD1**–**3** showed endothermic peaks due to *T*_g_ at 85°C, 57°C, and 65°C *T*_g_ at 85°C, 57°C, and 65°C, respectively, and then exothermic peaks due to *T*_c_ at 149°C, 126°C, and 125°C, respectively (Figure 28b). Therefore, the DSC traces for the ground solids are typical of an amorphous solid. Indeed, the XRD peaks of the ground solids after heating beyond *T*_c_ are similar to those before grinding, suggesting that heating restores the microcrystalline structure.

**FIGURE 29:**
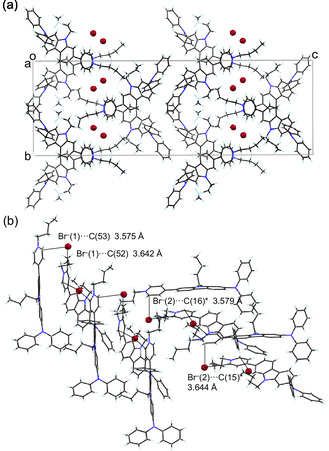
(a) Crystal packing and (b) a continuous intermolecular *π*‐stacking and the distances between bromide ion and pyridinium ring for **OD2** (CCDC Deposition Number 928592). Adapted with permission from ref. [75]. Copyright 2013 Elsevier.

The X‐ray crystal structure of **OD2** showed that a continuous intermolecular *π*‐stacking was formed between the dye molecules, and partial *π*‐overlap was observed between the pyridinium ring and the carbazole moiety of the neighboring dye molecule (Figure 29). Four short interatomic contacts of less than 3.6 Å were observed between a pair of dye molecules. The bromide ion is located close to the pyridinium rings in the pair of dye molecules, and the atomic distances between the bromide ion and the carbon atom of the pyridinium ring are ca. 3.58 and 3.64 Å, respectively. Thus, it was concluded that the interaction between the pyridinium ring and bromide ion and the continuous intermolecular *π*‐stacking among the dye molecules may cause the bathochromic shifts of *λ*_max_^abs^, the hypsochromic shift of *λ*_max_^fl^, and the decrease in Φ_fl_ value upon transition from the solution to the crystalline state.

Consequently, we concluded that the MFC of D*–π*–A‐type pyridinium fluorescent dyes having counter anions is due to the reversible switching between the crystalline state and the amorphous state, which is associated with the changes in intermolecular *π* *–π* interaction, dipole–dipole interaction, and especially the interaction between the pyridinium ring and counter anions, before and after grinding. We propose that the use of various counter anions for D*–π*–A pyridinium fluorescent dyes not only exhibits remarkable MFC but also controls the changes in fluorescence properties upon grinding or heating, which may be one of the most effective strategies for fine‐tuning the MFC‐induced fluorescence color change.

## 7 Molecular Design of D–*π*–A‐Type Mechanofluorochromic Dyes

As described in the above sections, we demonstrated that a newly designed and developed D*–π*–A‐type fluorescent dyes with a moderate dipole moment (*μ*_g _= ca. 5–6 Debye) in the ground state exhibit pronounced inter‐b‐MFC. Our study revealed that the inter‐b‐MFC of the D*–π*–A‐type fluorescent dyes is due to the reversible changes in not only intermolecular *π* *–π* interactions and dipole–dipole interactions but also the formation of intermolecular hydrogen bonds between the crystalline state before grinding and the amorphous state after grinding (Figure 19). It was suggested that the D*–π*–A‐type fluorescent dyes with a large dipole moment (*μ*_g _= ca. 8 Debye) exhibit weak MFC due to the formation of strong dipole–dipole interactions between the dye molecules that may inhibit the change in molecular arrangement between crystalline state and amorphous state by the grinding, preventing the expression of MFC. Furthermore, it was found that the weak MFC of D*–π*–A‐type fluorescent dyes with a small dipole moment (*μ*_g _= ca. 1−2 Debye) is attributed to small changes in the dipole–dipole interactions between the dye molecules in the crystalline state and amorphous state. Meanwhile, (D*–π*–)_2_A‐type fluorescent dyes with a moderate dipole moment (*μ*_g _= ca. 3 Debye) and ICT characteristics allow for the activation of MFC. Consequently, we proposed that the most important point for developing mechanofluorochromic dyes is to construct D*–π*–A structure with a moderate dipole moment (ca. 5 Debye in our D*–π*–A‐type fluorescent dyes). Furthermore, our work demonstrated the MFC of D*–π*–A‐type pyridinium fluorescent dyes having counter anions is due to the reversible switching between the crystalline state and the amorphous state, which is associated with the changes in intermolecular *π* *–π* interaction, dipole–dipole interaction, and especially the interaction between the pyridinium ring and counter anions, before and after grinding.

Therefore, we can see the structural characteristics of D*–π*–A‐type fluorescent dyes that are suitable for expressing and precisely tuning the MFC by systematically varying the molecular orientation and arrangement and intermolecular interactions between the dye molecules include the following: (1) the color and fluorescence color of the dye can be adjusted by changing the electron‐donating group (D), the electron‐withdrawing group (A), and the*π*‐conjugated bridge; (2) the magnitude of the dipole moment, that is, the dipole–dipole interaction can be adjusted by changing D and A; (3) the *π* *–π* interaction between dye molecules can be controlled by changing the steric hindrance of the substituents; (4) intermolecular hydrogen bonds can be controlled by replacing the substituents on heteroatoms (nitrogen and oxygen atoms) from hydrogen to alkyl groups or by converting carboxyl groups to ester groups; and (5) the intermolecular *π* *–π* interaction and the dipole–dipole interaction can be adjusted by exchanging the counter anions of pyridinium ring as the A moiety.

In this way, by tuning D*–π*–A system by changing the electron‐donating ability of D moiety, the electron‐withdrawing ability of A moiety, the steric size of substituents, and the magnitude of *π*‐conjugated bridge (fluorophore), it is possible to freely control the intermolecular dipole–dipole and *π* *–π* interactions, intermolecular hydrogen bonds, and molecular orientation and arrangement between the dye molecules. Thus, we not only clarified the MFC of the D*–π*–A‐type fluorescent dye but also provided the molecular design direction for creating new D*–π*–A‐type mechanofluorochromic dyes.

## 8 Summary and Outlook

In this Personal Account, we have presented that D*–π*–A‐type fluorescent dyes with moderate dipole moments exhibit inter‐MFC attributed to reversible changes in dipole–dipole interactions, intermolecular hydrogen bonds, and intermolecular *π* *–π* interactions between the crystalline state before grinding and the amorphous state after grinding. Moreover, our work suggested that D*–π*–A‐type fluorescent dyes possessing crystal polymorphism and D*–π*–A‐type cationic fluorescent dyes bearing counter anion are expected to express h‐MFC as well as pronounced b‐MFC. Indeed, for the b‐MFC based on the dipole moment of dyes, Zhang et al. discovered that tetraphenylethylene (TPE) derivatives with large dipole moments and strong ICT characteristics are densely packed in the amorphous solids through the formation of strong dipole–dipole interactions, but upon grinding, the ICT characteristics are enhanced by the increased molecular planarity, resulting in a remarkable b‐MFC [17]. In contrast, the TPE derivatives with small dipole moments and weak ICT characteristics exhibit unapparent MFC due to their weak dipole–dipole interactions. Meanwhile, Thomas III et al. demonstrated that D*–π*–A‐type phenylene ethynylenes (PEs) with a strong D unit show b‐MFC attributed to the formation of planarized/aggregated PEs upon grinding. On the other hand, the PEs with a relatively weak D unit show h‐MFC attributed to the destruction of PE aggregates upon grinding [76, 77–78]. Misra et al. reviewed that D–A‐type fluorescent dyes, including triphenylamine‐, TPE‐, and benzothiadiazole‐based derivatives, exhibit h‐MFC [8]. On the other hand, Yamanoi et al. revealed that a disilane‐bridged D–A–D triad consisting of two phenothiazine units as D moiety and a thienopyrazine unit as A moiety exhibits b‐MFC due to the change in conformational relaxation, which is caused by the flexibility of the Si—Si bond and the phenothiazine group, between the crystalline state and amorphous state [95]. Yin et al. found that a symmetric A–D–A‐type aggregation‐induced emission (AIE)‐active luminogen which consists of two triphenylamine groups as D moiety and two (trifluoromethyl)benzene groups as A moiety integrated into the central fluorene skeleton exhibits hypso‐ and bathochromic bidirectional fluorescence mechanochromisms accompanied by a large shift of fluorescence emission wavelength over 120 nm [96]. Furthermore, they demonstrated that the tricolored fluorescence mechanoresponsiveness is attributed to the morphological transformation of crystalline‐to‐amorphous states and the crystalline‐to‐crystalline phase transition.

More recently, Sun et al. reported the remarkable AIEE and high‐contrast MCL behaviors D–A‐type phenothiazine‐functionalized cyclic chalcone derivative exhibits and demonstrated their advanced application in high‐sensitivity latent fingerprint visualization, dynamic anticounterfeiting systems, and multilevel optical encryption platforms [90]. Consequently, D*–π*–A‐type fluorescent dyes can exhibit b‐MFC, which originates from the distortion or twist between the *π*‐conjugated bridge and the D or A moiety and/or h‐MFC, which originates from the change in the intermolecular*π* *–π* interactions and dipole–dipole interactions between the dye molecules.

The MFC of organic fluorescent dyes is important for developing the research field of dynamic solid‐state luminescence chemistry and for creating dynamic luminescent molecular devices and thus has received considerable attention in recent years. By promoting MFC research among many researchers in fields including organic chemistry, photochemistry, electrochemistry, computational chemistry, analytical chemistry, and photophysics, we believe to deepen our comprehensive understanding of the expression of MFC, which will confirm a molecular design direction for creating mechanofluorochromic dyes that exhibit not only b‐MFC but also h‐MFC, leading to development of epoch‐making molecular luminescent materials and their devices based on MFC.

## Funding

This work was supported by the Japan Society for the Promotion of Science (JSPS) KAKENHI; 18H04520; 23K18521; 25K01808.

## Conflicts of Interest

The author declare no conflicts of interest.

## Data Availability

The data that support the findings of this study are available from the corresponding author upon reasonable request.
